# Supplementary material for: Enhanced Malignant Phenotypes of Glioblastoma Cells Surviving NPe6-Mediated Photodynamic Therapy are Regulated via ERK1/2 Activation
Source: Cancers (Basel). 2020 Dec 4;12(12):3641. doi: 10.3390/cancers12123641 (PMC7761910; doi:10.3390/cancers12123641)

Figure 1B: T98G Cleaved PARP

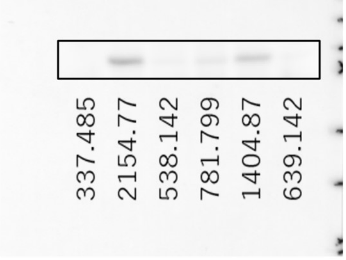

Figure 1B: U343MG Cleaved PARP

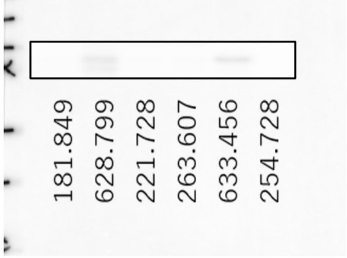

Figure 1B: T98G GAPDH

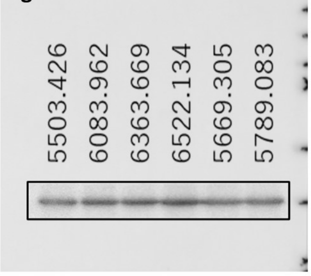

Figure 1B: U343MG GAPDH

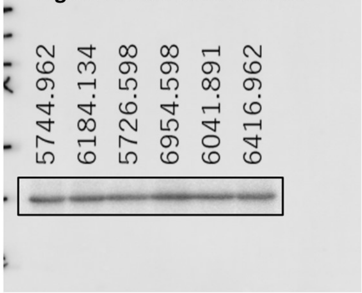

Figure 1D: T98G Bax crosslinker+

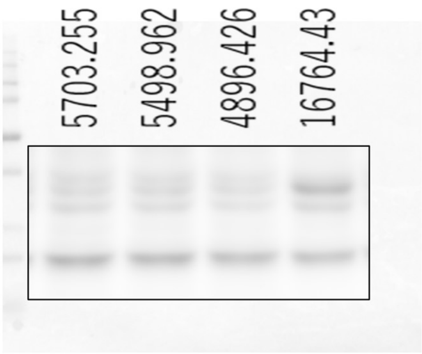

Figure 1D: U343G Bax crosslinker+

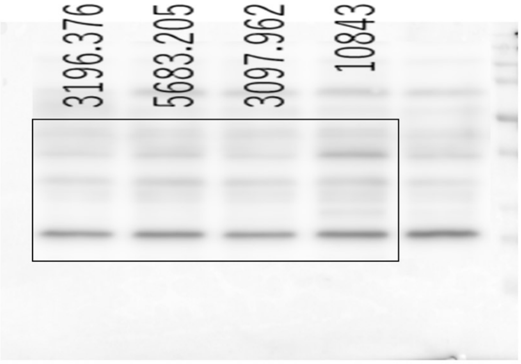

Figure 1D: T98G Bax crosslinker-

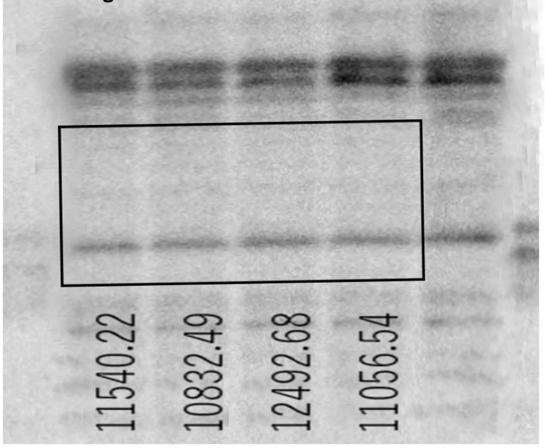

Figure 1D: U343MG Bax crosslinker-

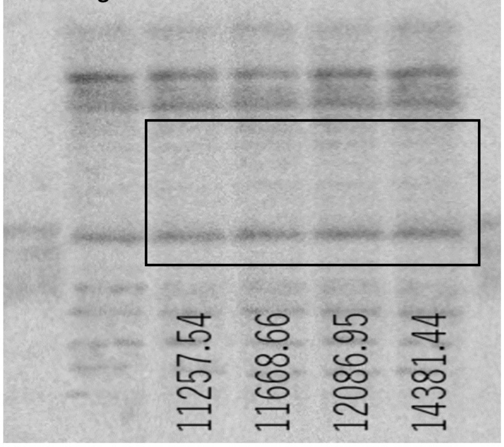

Figure 2A: T98G PAR

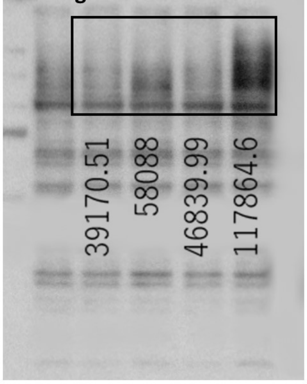

Figure 2A: U343MG PAR

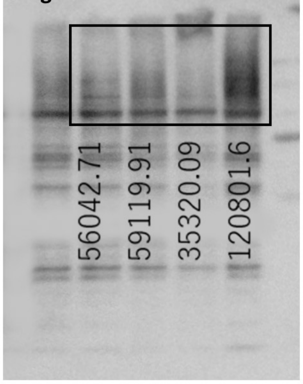

Figure 2A: T98G GAPDH

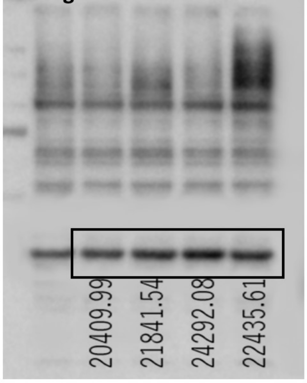

Figure 2A: U343MG GAPDH

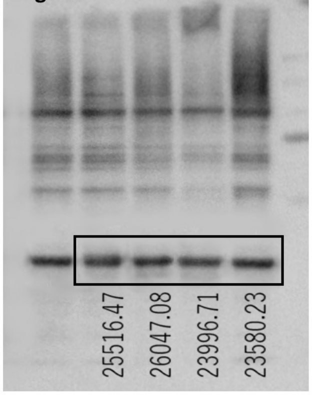

Figure 2C: T98G PAR

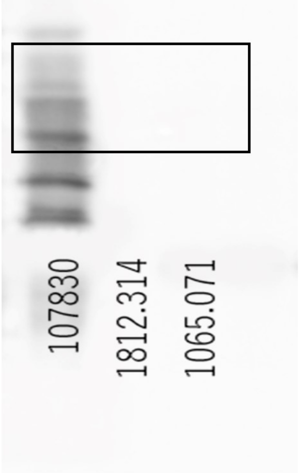

Figure 2C: U343MG PAR

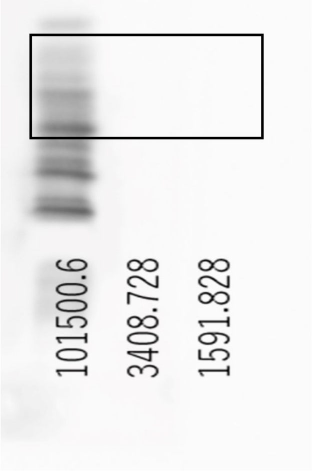

Figure 2C: T98G GAPDH

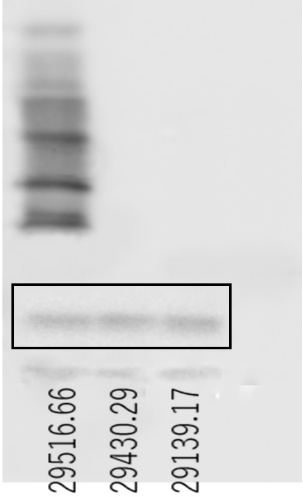

Figure 2C: U343MG GAPDH

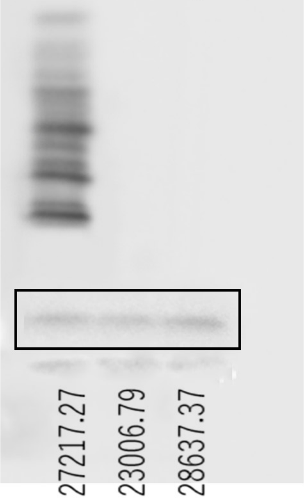

Figure 2C: T98G Cyt C (mitochondria)

Figure 2C: U343MG Cyt C (mitochondria)

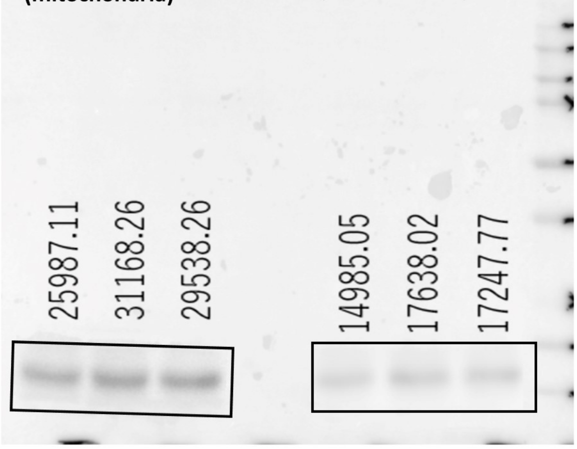

Figure 2C: T98G AIF (mitochondria)

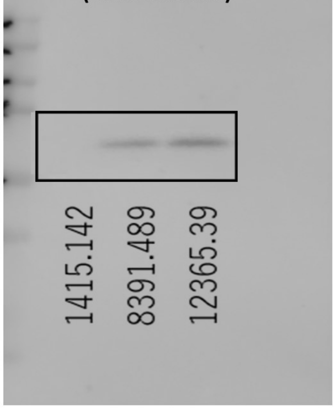

Figure 2C: U343MG AIF (mitochondria)

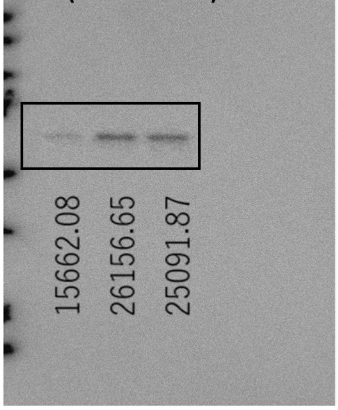

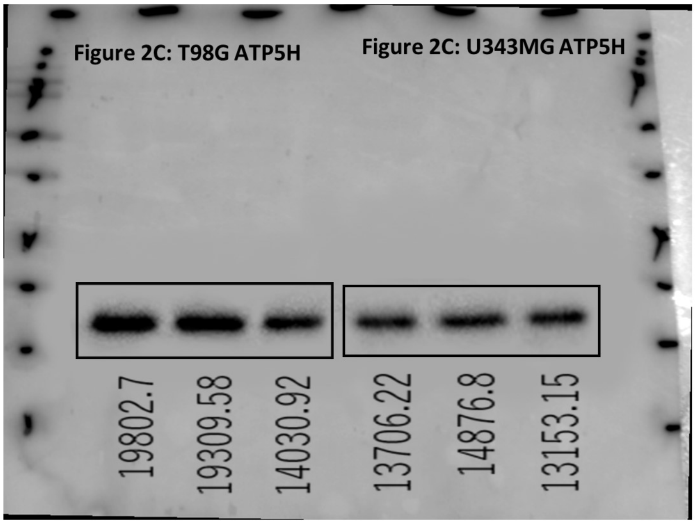

Figure 2C: T98G Cyt C (cytosol)

Figure 2C: U343MG Cyt C (cytosol)

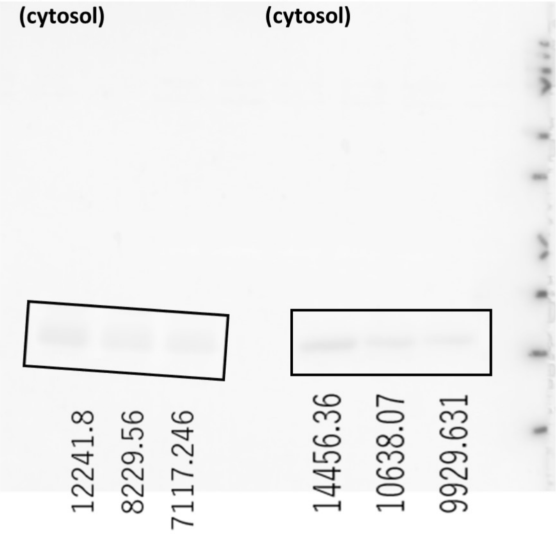

Figure 2C: T98G AIF (nuclear)

Figure 2C: U343MG AIF (nuclear)

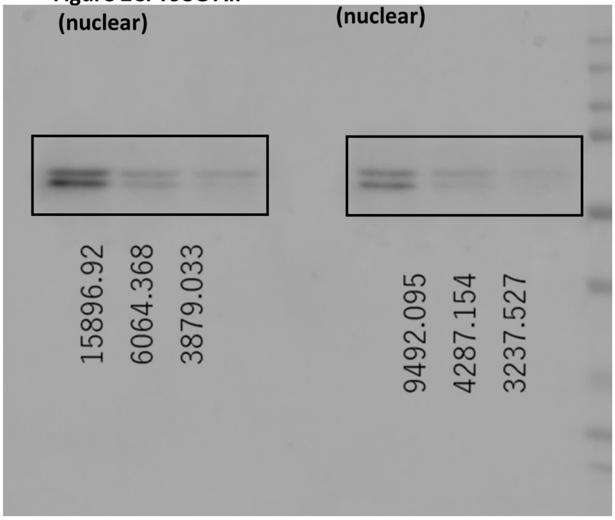

Figure 2C: T98G GAPDH

Figure 2C: U343MG GAPDH

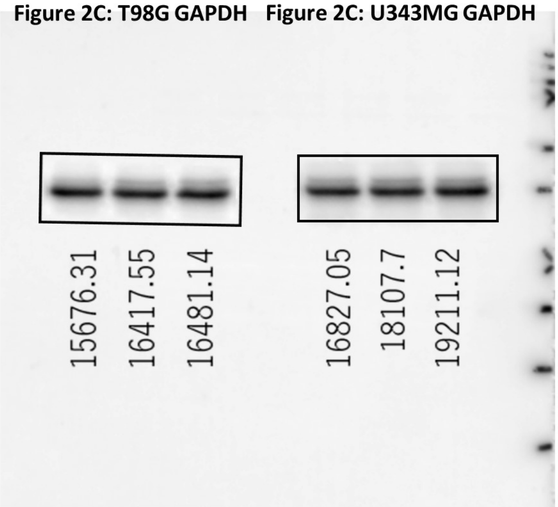

Figure 2C: T98G Myc (nuclear)

Figure 2C: U343MG Myc (nuclear)

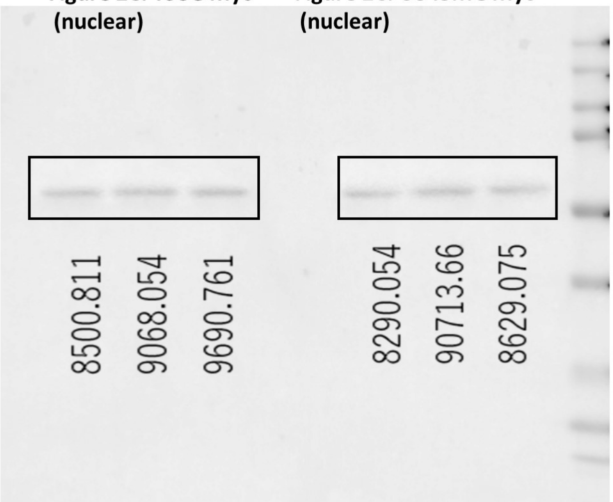

Figure 3a:
T98G Bax

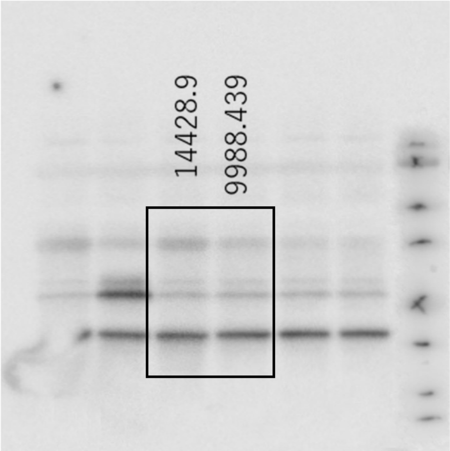

Figure 3a:
U343MG Bax

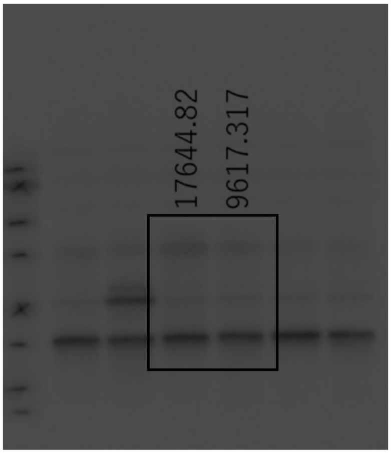

Figure 3a:
U343MG Cle-PARP

Figure 3a:
T98G P-ERK1/2

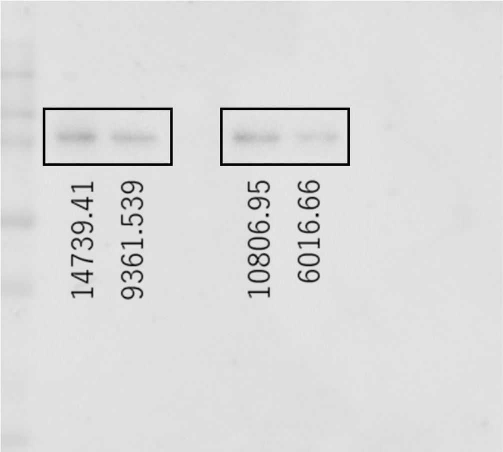

Figure 3a:
U343MHG GAPDH

Figure 3a:
T98G GAPDH

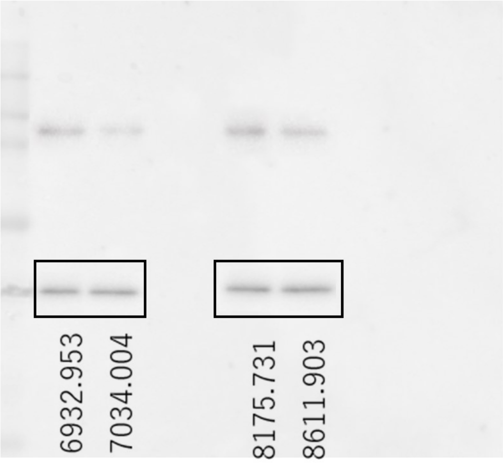

Figure S3:
T98G P-ERK1/2

Figure S3:
U343MG P-ERK1/2

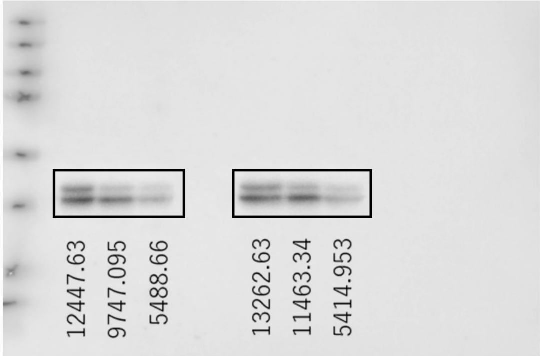

Figure S3:
T98G GAPDH

Figure S3:
U343MG GAPDH

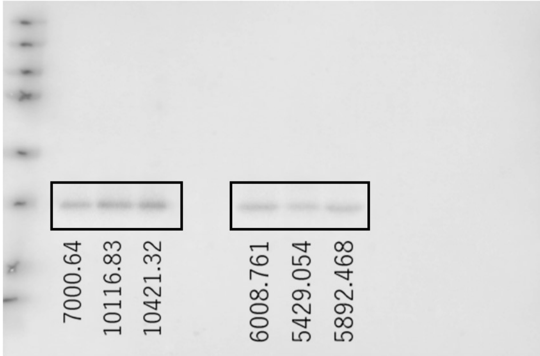

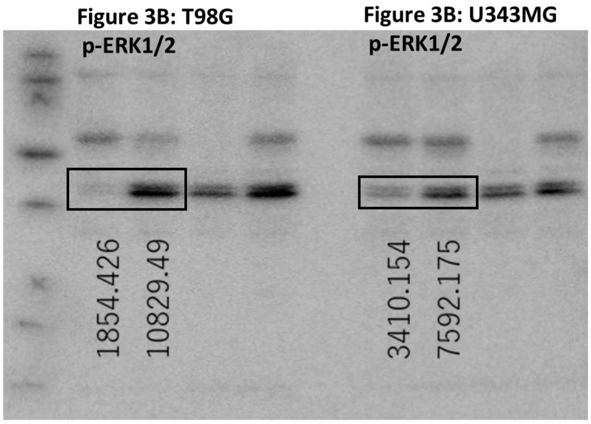
Figure 3B: T98G
p-ERK1/2
Figure 3B: U343MG
p-ERK1/2
1854.426
10829.49
3410.154
7592.175

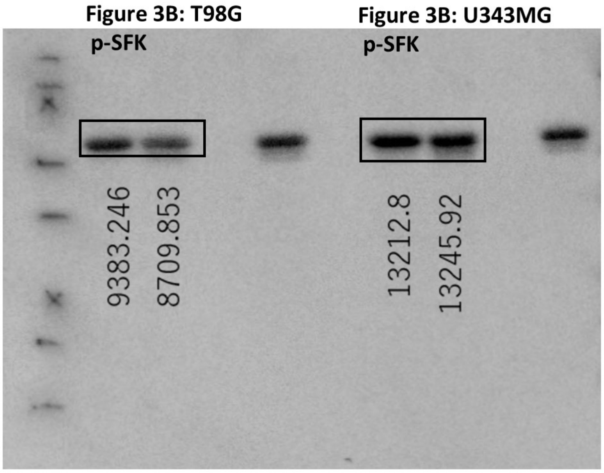
Figure 3B: T98G
p-SFK
Figure 3B: U343MG
p-SFK
9383.246
8709.853
13212.8
13245.92

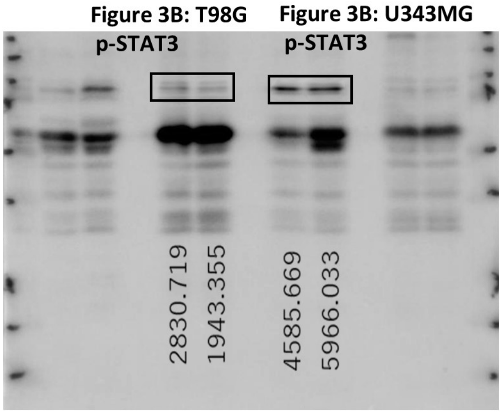
Figure 3B: T98G
p-STAT3
Figure 3B: U343MG
p-STAT3
2830.719
1943.355
4585.669
5966.033

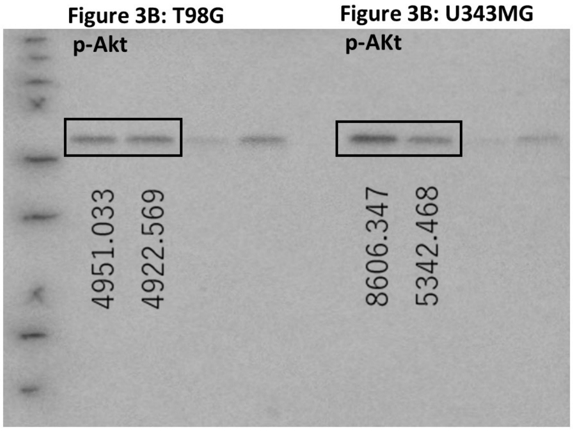
Figure 3B: T98G
p-Akt
Figure 3B: U343MG
p-AKt
4951.033
4922.569
8606.347
5342.468

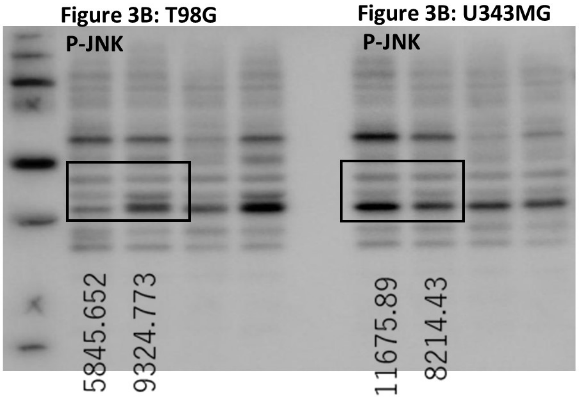
Figure 3B: T98G
P-JNK
Figure 3B: U343MG
P-JNK
5845.652
9324.773
11675.89
8214.43

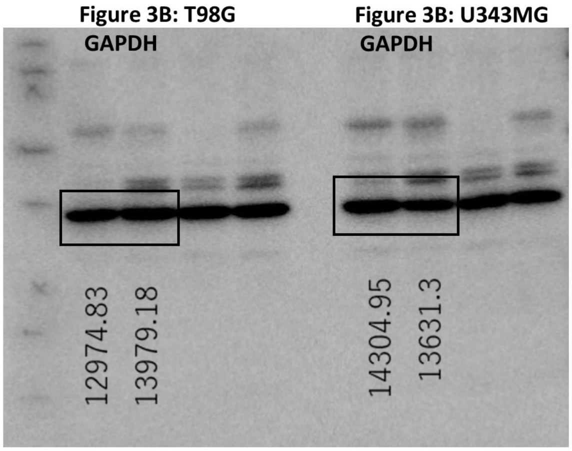
Figure 3B: T98G
GAPDH
Figure 3B: U343MG
GAPDH
12974.83
13979.18
14304.95
13631.3

Figure 3C: T98G Bax crosslink

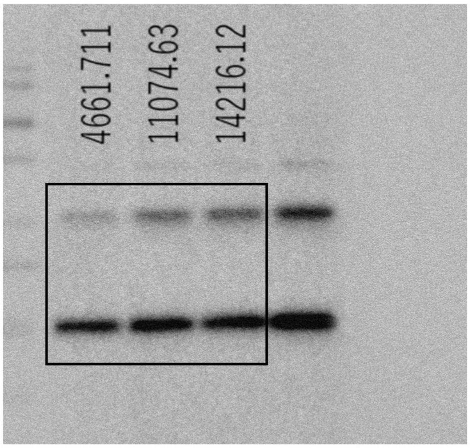

Figure 3C: U343MG Bax crosslink

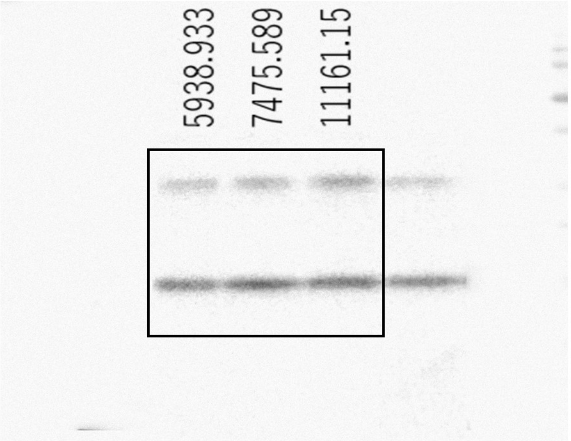

Figure 4B: T98G P-ERK1/2

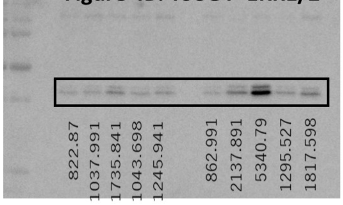

Figure 4B: U343MG P-ERK1/2

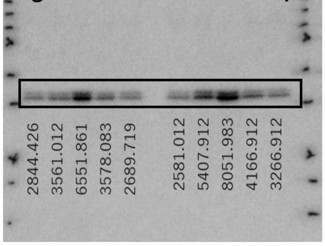

Figure 4B: T98G total-ERK1/2

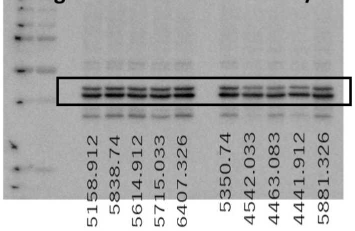

Figure 4B: U343MG total-ERK1/2

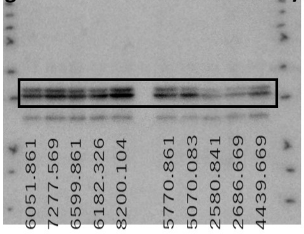

Figure 4B: T98G p-Akt **Figure 4B: U343MG p-Akt**

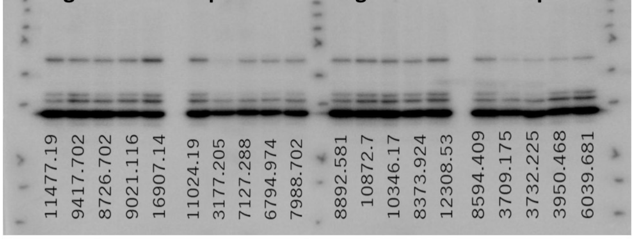

Figure 4B: T98G total-Akt **Figure 4B: U343MG total-Akt**

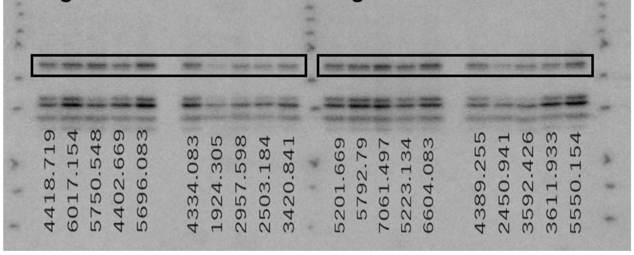

Figure 4B: T98G p-STAT3 **Figure 4B: U343MG p-STAT3**

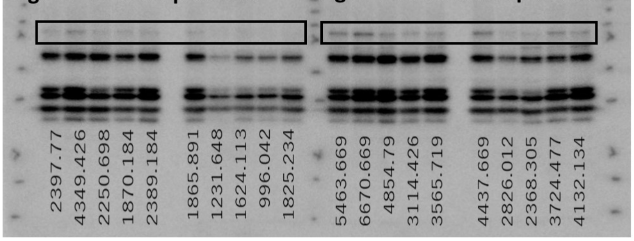

Figure 4B: T98G total-STAT3 **Figure 4B: U343MG total-STAT3**

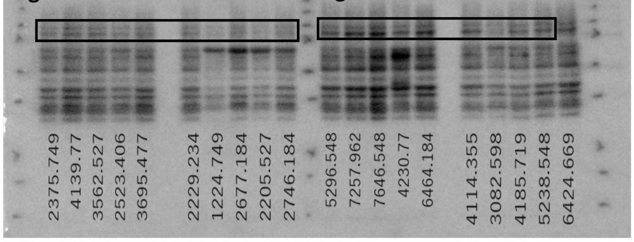

Figure 4B: T98G P-SFK **Figure 4B: U343MG P-SFK**

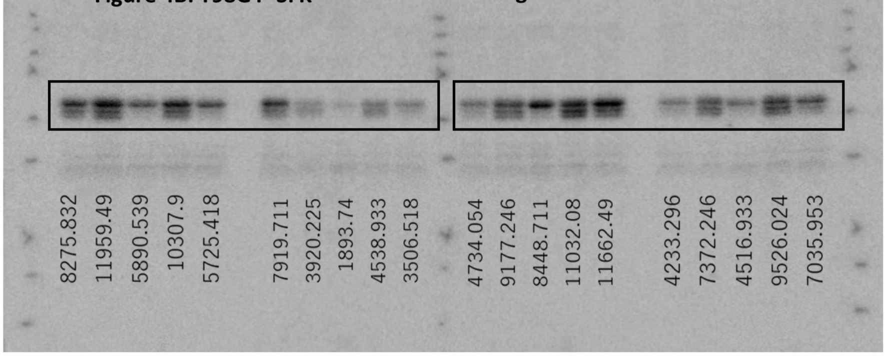

Figure 4B: T98G toal c-SRC **Figure 4B: U343MG toal c-SRC**

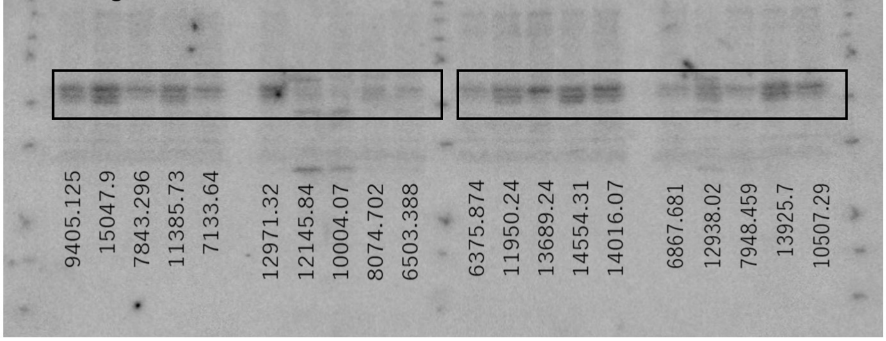

Figure 4B: T98G GAPDH **Figure 4B: U343MG GAPDH**

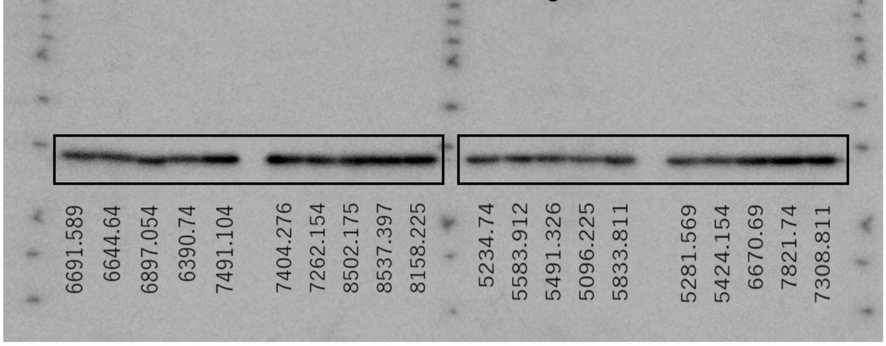

Figure 4C: T98G P-ERK1/2 **Figure 4C: U343MG P-ERK1/2**

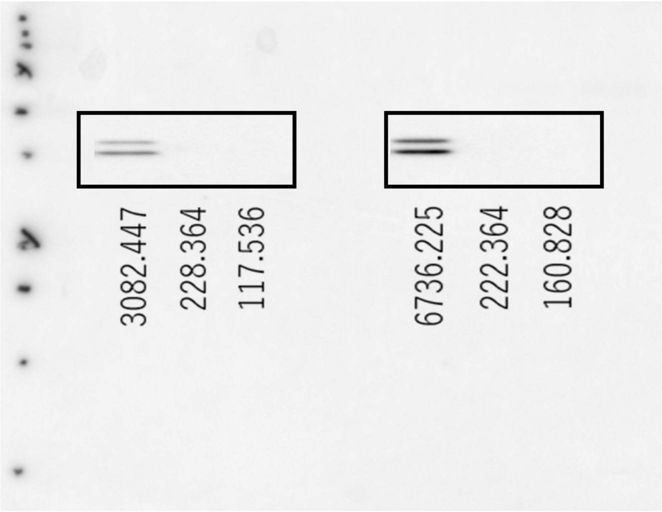

Figure 4C: T98G GAPDH **Figure 4C: U343MG GAPDH**

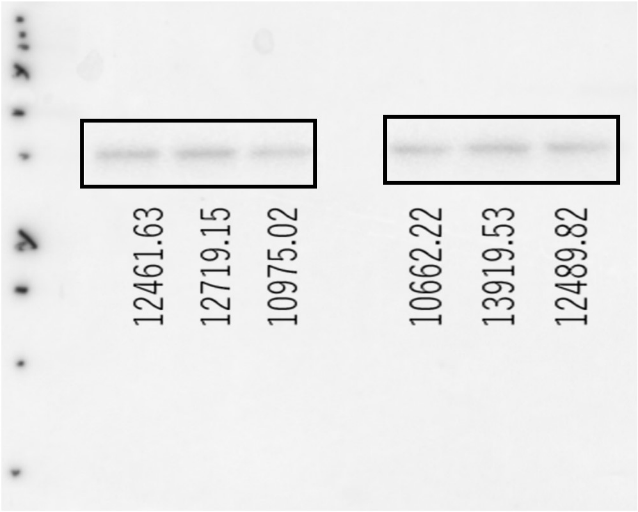

Figure S2: T98G whole -Bax

Figure S2: U343G whole -Bax

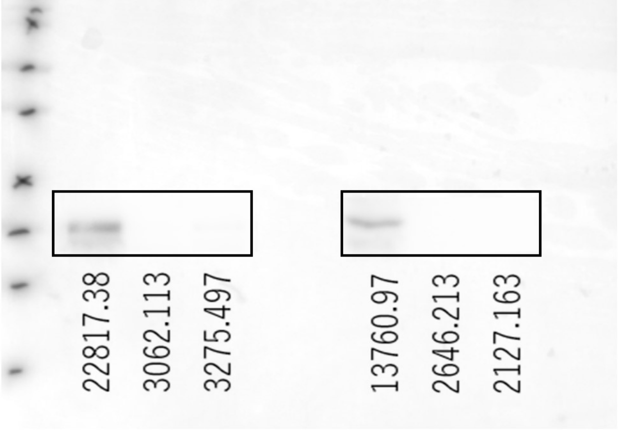

Figure S2: T98G whole -Bak

Figure S2: U343G whole -Bak

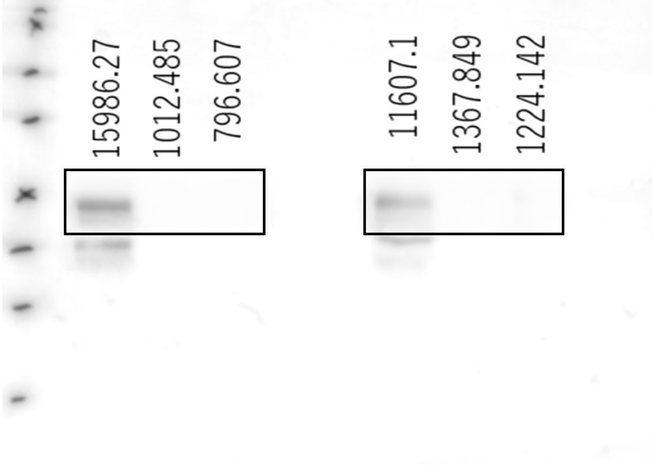

Figure S2: T98G whole -GAPDH

Figure S2: U343G whole -GAPDH

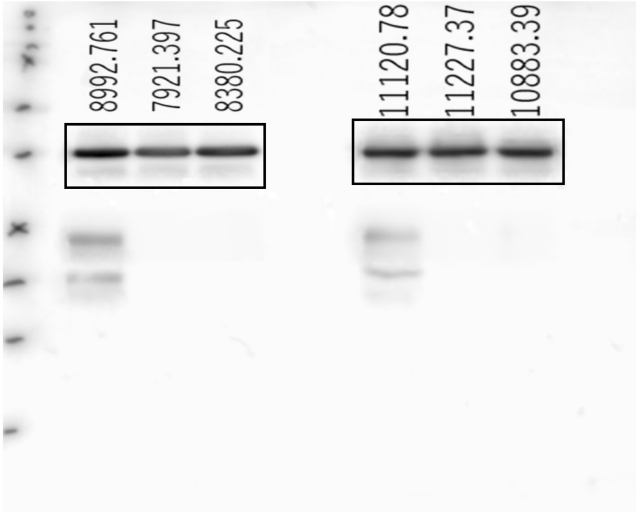

Figure S2: T98G
Cyt C

Figure S2: U343G
Cyt C

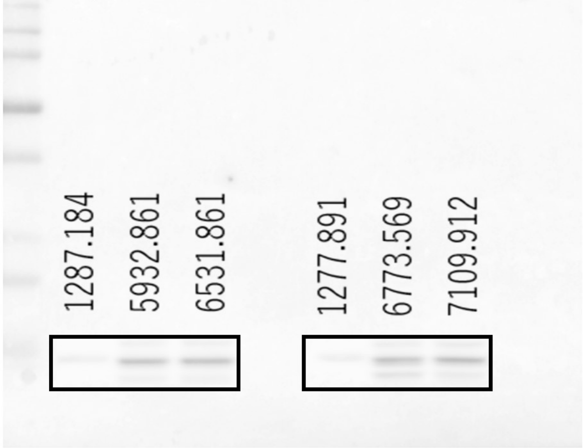

Figure S2: T98G
AIF

Figure S2: U343G
AIF

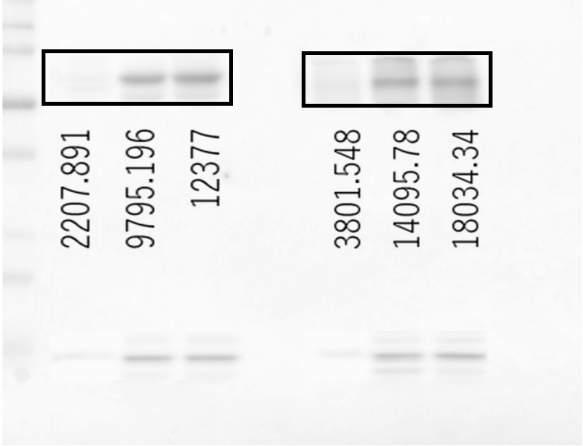

Figure S2: T98G
ATP5H

Figure S2: U343G
ATP5H

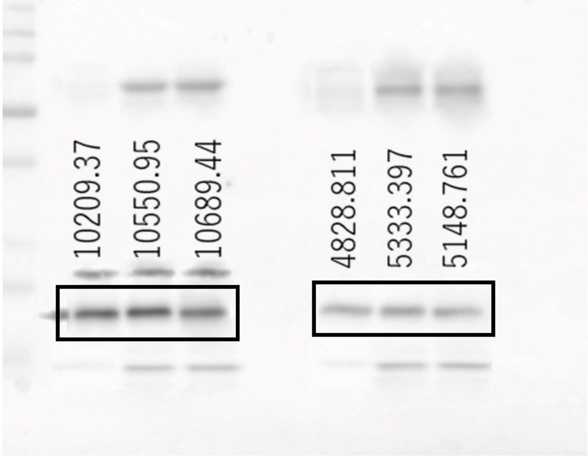

Figure S4 CD44

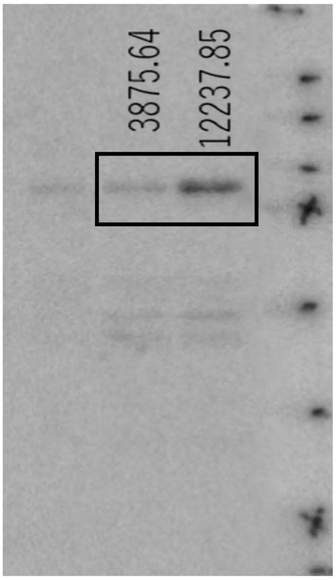

Figure S4 Actin

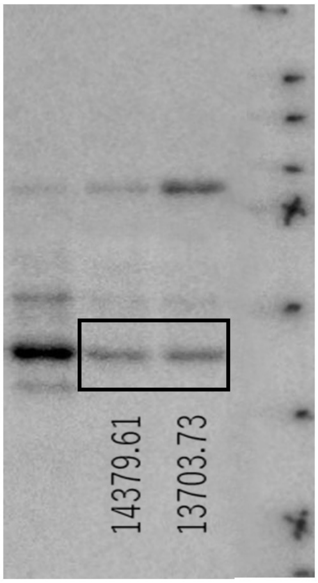

Supplement: Supplementary file 1 [file cancers-12-03641-s001.zip › cancers-979198_supplementary/cancers-979198_raw WB.pdf]
