# Supplementary material for: Enhanced Malignant Phenotypes of Glioblastoma Cells Surviving NPe6-Mediated Photodynamic Therapy are Regulated via ERK1/2 Activation
Source: Cancers (Basel). 2020 Dec 4;12(12):3641. doi: 10.3390/cancers12123641 (PMC7761910; doi:10.3390/cancers12123641)
Supplement: Supplementary file 1 [file cancers-12-03641-s001.zip › cancers-979198_supplementary/cancers-979198_supplementary.docx]

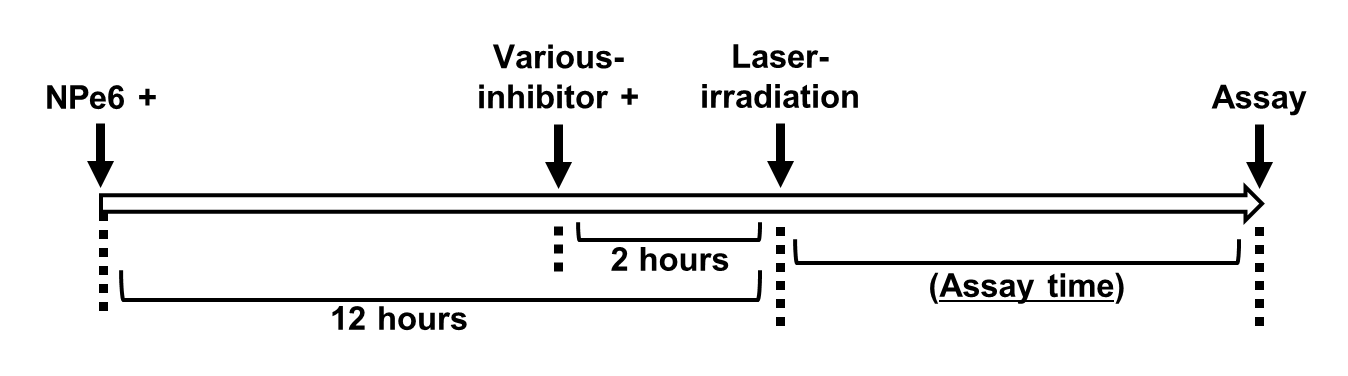


**Figure S1.** Schematic illustration of *in vitro* NPe6-PDT model. For details, see the Materials and Methods.


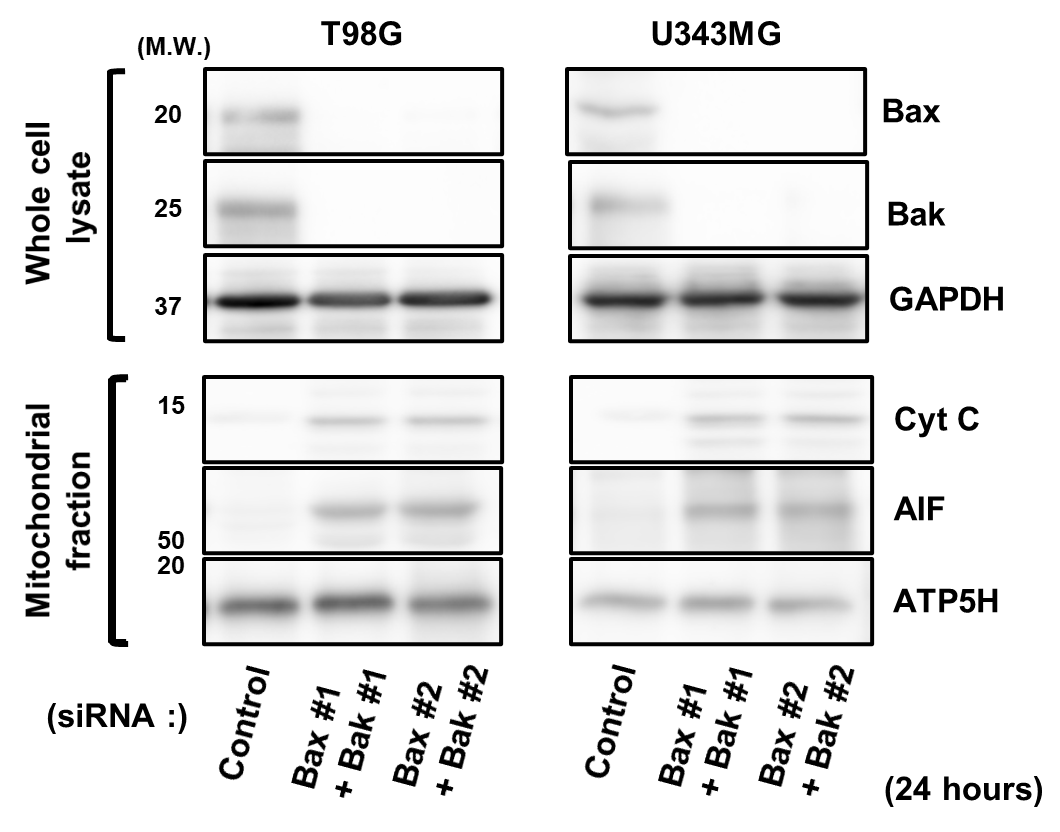


**Figure S2.** Dual knockdown of Bax and Bak completely blocked NPe6-PDT-induced intracellular translocation of AIF and cytochrome c in GBM cells. T98G and U343MG cells were treated with siRNAs as indicated. After 48 h, these cells were further subjected to NPe6-PDT (NPe6, 15 μg·mL^-1^) in the presence or absence of pretreatment with vehicle, 3-ABA (3 mM), or PJ34 (15 μM), as shown in the figure, and further cultured for 24 h. Then, the fractionated lysates of these treated cells were analyzed by immunoblotting using the indicated primary antibodies. Antibodies against GAPDH (cytosolic fraction marker) and ATP5H (mitochondrial fraction marker) were used to confirm the amount of proteins loaded in each fraction.


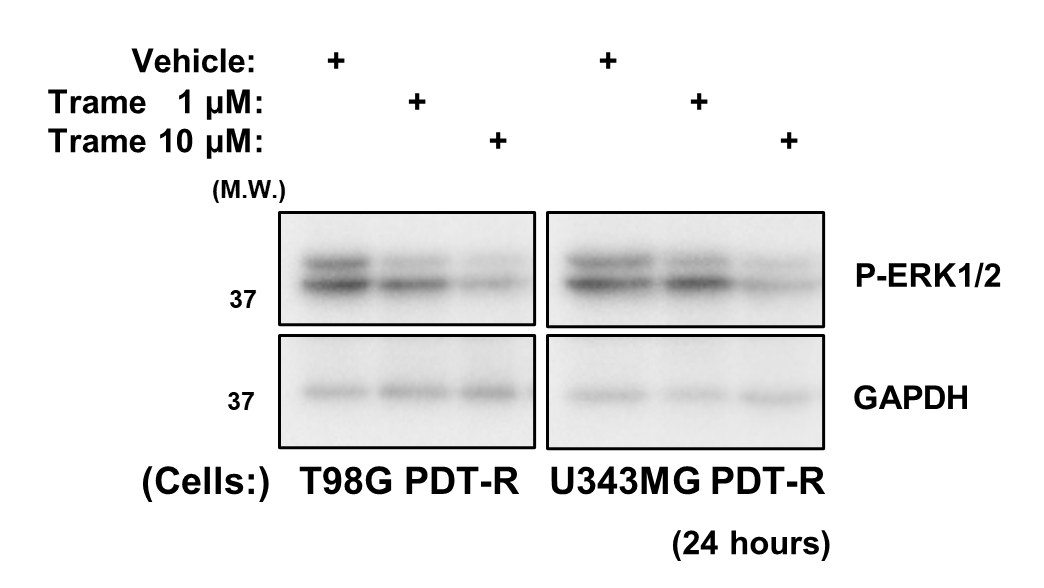


**Figure S3.** Trametinib blocked ERK1/2 phosphorylation (activation) in NPe6-PDT-R GBM cells by dose-dependent manner of trametinib. NPe6-PDT-R T98G and U343MG cells were treated with the vehicle or the indicated dose of the MEK1/2 inhibitor trametinib (Trame). After 24 h, the lysates of these treated cells were analyzed by immunoblotting using the indicated primary antibodies. GAPDH antibodies were used to confirm the amounts of protein loaded.


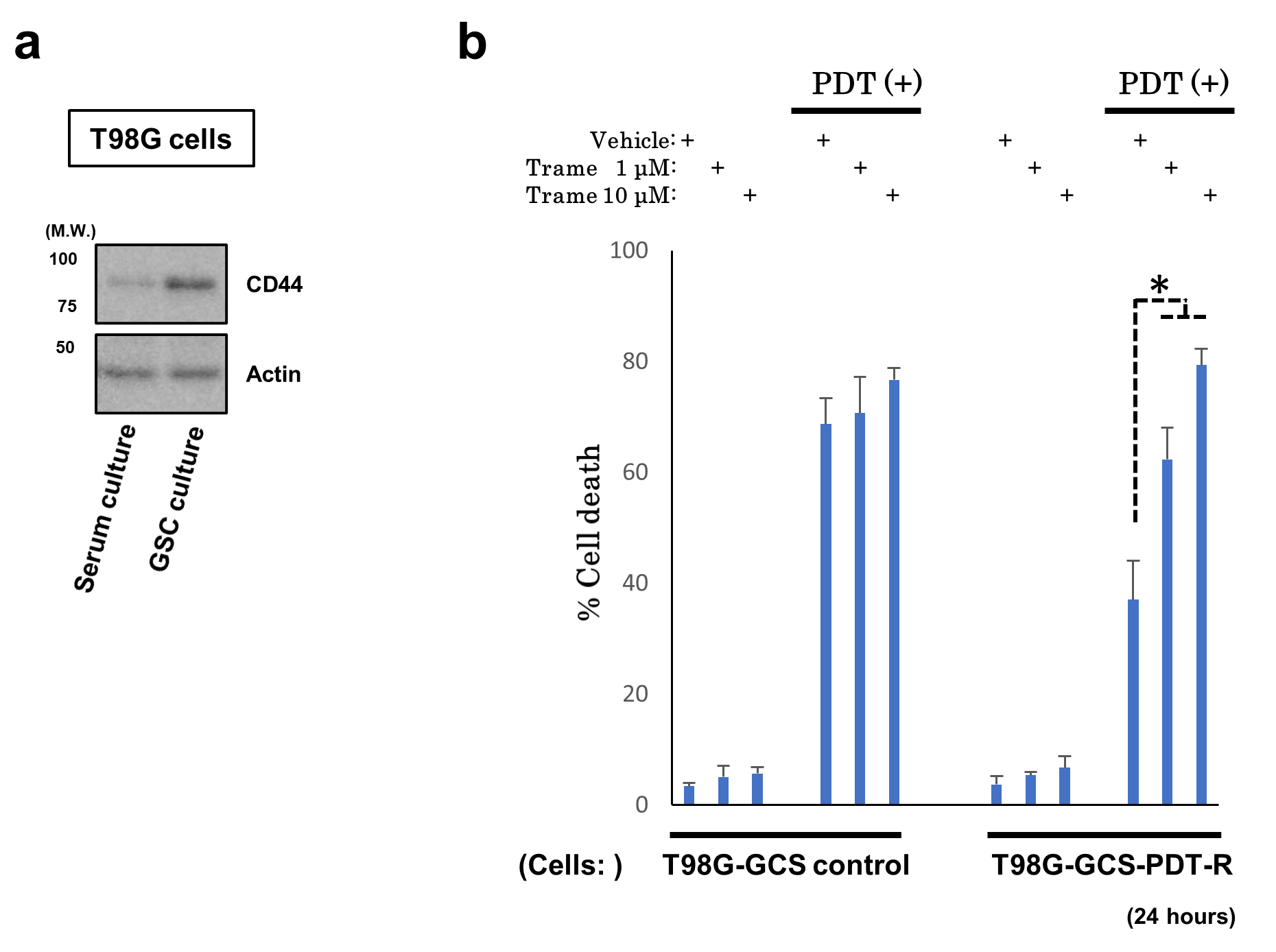


**Figure S4.** Trametinib reversed the resistance against PDT-induced cell death of the glioma stem cells of T98G cells (T98G-GCSs) survived from PDT. (**a**) The cell lysates of original T98G cells (Serum culture) and T98G-GCSs (GSC culture, see supplementary materials and methods) cultured by monolayer on the type I collagen-coated dish were analyzed by immunoblotting using the primary antibody of CD44, one of the stem cell markers of GBM, and GAPDH as shown in the figures. GAPDH antibodies were used to confirm the amounts of protein loaded. (**b**) T98G-GCSs and PDT-survived T98G-GSCs (T98G-GSC-PDT-Rs, see supplementary materials and methods) cultured by monolayer on the type I collagen-coated dish were subjected to NPe6-PDT (NPe6, 5 μg·mL^-1^) in the presence or absence of pretreatment with the vehicle or the indicated dose of trametinib, as shown in the figure. After 24 h, the cell death rate of all treated cells was determined. **p*<0.01.


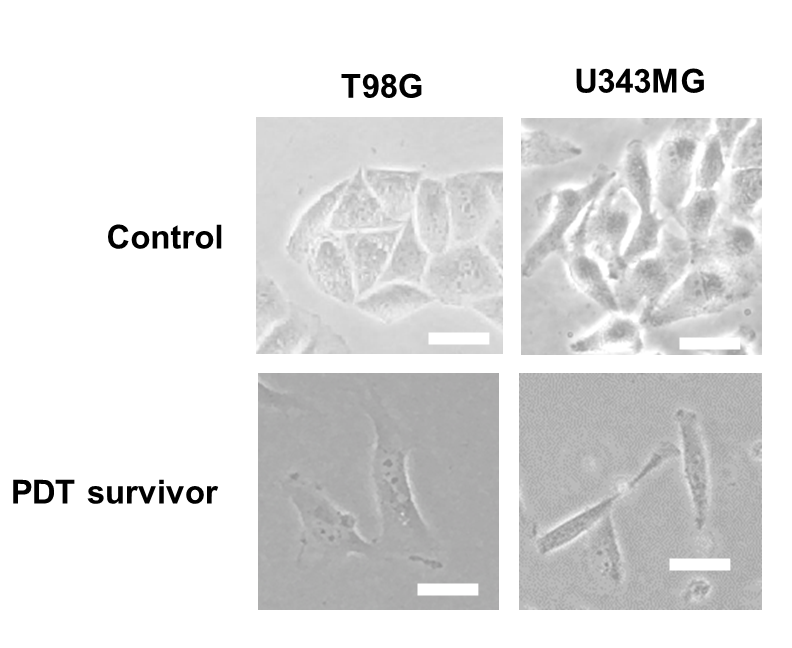


**Figure S5.** The morphological appearance of NPe6-PDT-R cells and their control cells. Control and NPe6-PDT-R T98G and U343MG cells were photographed under a phase-contrast microscope (×400). Scale bar, 10 μm.

Supplementary materials and methods

Establishment of the glioblastoma (GBM) stem cells of T98G cells (T98G-GCSs)

T98G cells were cultured by monolayer on the type I collagen-coated dish (AGC TECHNO GLASS, Shizuoka, Japan) under the serum-free GBM sphere culture medium supplemented with epidermal growth factor and fibroblast growth factors we previously described [1]. After 14 days by serial passage, the obtained cells were estimated as T98G-GCSs after confirming elevated expression of CD44, one of the stem cell markers of GBMs, by immunoblotting compared with the control T98G cells cultured under RPMI1640 medium supplemented with 10 % FBS and 1 % PS.

Establishment of the NPe6-PDT-survived T98G-GCSs

The NPe6-PDT-survived T98G-GCSs were established same as the case of the control T98G cells with the exception of NPe6 dose (5 µg·mL^-1^).

Reference

1. Sato A, Sunayama J, Okada M, et al. Glioma‐initiating cell elimination by metformin activation of FOXO3 via AMPK. Stem Cells Transl Med. 2012;1:811‐824.
